# Fruit fly infestation of cucurbitaceous vegetables in Morogoro—Eastern Central Tanzania

**DOI:** 10.1371/journal.pone.0322277

**Published:** 2025-04-21

**Authors:** Petronila Tarimo, Sija Kabota, Ramadhan Majubwa, Abdul Kudra, Massimiliano Virgilio, Kurt Jordaens, Marc De Meyer, Maulid Mwatawala

**Affiliations:** 1 Department of Crop Science and Horticulture, Sokoine University of Agriculture (SUA), Morogoro, Tanzania; 2 Research, Consultancy and Publication Unit, National Sugar Institute (NSI), Morogoro, Tanzania; 3 Royal Museum for Central Africa, Invertebrates Section and JEMU, Tervuren, Belgium; DIU: Dhaka International University, BANGLADESH

## Abstract

Fruit flies (Diptera: Tephritidae) are a significant threat to cucurbit production in Tanzania. To effectively combat these pests, it is crucial to understand the patterns of fruit fly infestations among cucurbit crops at different altitudes. Our study focused on the infestation patterns of fruit flies among commonly grown cucurbit crops—cucumber (*Cucumis sativus* L.), watermelon (*Citrullus lanatus* [Thunb.] Matsum. & Nakai), and squash (*Cucurbita moschata* D.) in Eastern Central Tanzania. The research was conducted in the “plateau” (low altitude) and the “mountainous” (high altitude) areas of Morogoro over three cropping seasons (March - April as Season I, June – July as Season II, and September – October as Season III) during 2020. We collected a total of 450 samples equivalent to 4,500 fruits during this period. Out of these, 418 samples were infested by five fruit fly species: *Zeugodacus cucurbitae* (Coquillett), *Dacus ciliatus* Loew, *Dacus vertebratus* Bezzi*, Dacus bivittatus* (Bigot), and *Dacus punctatifrons* Karsch, which are the principal tephritid pests of cucurbit crops in Morogoro. In total, 22,169 fruit flies were recovered from the samples, with more flies emerging from the mountainous zone (12,390) than the plateau (9,779). *Zeugodacus cucurbitae* accounted for the majority of the emerged flies (18,789), while the remaining species, including *D. vertebratus* and *D. ciliatus,* contributed smaller numbers. Among the hosts, *Cucumis sativus* was the most heavily infested, followed by *Cucurbit moschata* and *Citrullus lanatus.* Significant effects of fruit fly species × host species and fruit fly species × agroecological zone on percent infestation and degree of infestation. Furthermore, the effects of host species × agroecological zone on percent infestation were significant. The study further found that that *Z. cucurbitae* was the dominant pest in terms of percent and degree of infestation among the three cucurbit crops at both agroecological zones in the Morogoro region. These findings provide valuable insights into severity of pest infestation that may cause high economic losses in cucurbit crops in cucurbit production. The study recommends that any management strategies for fruit flies should focus on controlling *Z. cucurbitae*, as a key pest of cucurbits in the region.

## 1. Introduction

Cucurbitaceous crops (Cucurbitales: Cucurbitaceae) are among the most important vegetable crops grown worldwide for their nutritional and economic value [1]. In sub-Saharan Africa, cucurbit crops are of considerable economic importance for local diet, employment, and income generation [2,3]. The commonly grown cucurbit species in Africa include cucumber (*Cucumis sativus* L.), watermelon (*Citrullus lanatus* [Thunb.] Matsum. & Nakai), and squash (*Cucurbita moschata* D.) [2,4]. Despite their economic importance, cucurbit crop production in Africa faces several challenges, including insect pest infestation, diseases, and climate change.

Fruit flies (Diptera: Tephritidae) pose a significant threat to cucurbit production in Africa, causing yield losses of up to 100% [5–7] depending on environmental factors and the susceptibility of crop species [8]. In Tanzania, the impact of fruit flies on cucurbit crops has not been quantified, yet significant losses are reported by farmers [9–11]. These losses stem from the oviposition activities of mature female flies, which puncture the delicate outer skin of immature cucurbit fruits to lay their eggs [12–14], leading to visible damage and creating tunnels conducive to fungal infections [13]. This damage not only reduces the aesthetic and commercial value of fruits but also impacts their marketability in local and external markets [3,10,15]. Despite the considerable efforts made by governmental entities, research organisations, and individuals to implement programs aimed at controlling and monitoring the spread and impact of fruit flies in Africa [16–20], several African nations continue to experience huge yield losses due to a lack of adequate knowledge for managing fruit flies [7,9,19].

Fruit fly infestation intensity can vary based on crop species and altitude [20,21]. Crop species exhibit differences in nutritional, structural, and chemical composition [3,22,23], which can significantly influence infestation levels [15,24,25]. A thorough understanding of the patterns of fruit fly infestation dynamics across different host species is imperative and can aid valuable information for developing targeted and effective fruit fly management strategies [12,16,26–29].

A diversity of fruit flies infesting cucurbits in Africa has been explored to a considerable extent (for example De Meyer et al.[30,31], Mwatawala et al.[9,[16]–18], Tanga and Rwomushana [32], Vayssières et al. [33]). Most available studies are limited to host range and preference, primarily focusing on the invasive *Zeugodacus cucurbitae* (Coquillett) [3,18,34]. Only a few studies, for example, Kambura et al. [12] and Mokam et.[3] concentrated on other prevalent fruit fly species, including *Dacus bivittatus* (Bigot), *Dacus ciliatus* Loew, *Dacus punctatifrons* Karsch, and *Dacus vertebratus* Bezzi. Previous studies in East Africa reported dominance of *Z. cucurbitae* over native species over most vegetable crops [9,12, 18,32] followed by *D. cilatus* which predominates on some hosts. In the presence of *Z. cucurbitae,* the other species of tephritid flies attacking cucurbits can be ranked as moderate, and are localized in their distribution causing varying degrees of infestation on vegetables depending on the season, variety and agroecology[32]. Contrasting results were reported from West Africa where *D. vertebratus* dominated *Z. cucurbitae* and other native cucurbit infesters [4,35]. However, information such as Degree of Infestation (number of emerged adults per unit weight of fruits) and Percentage Infestation (percentage of infested samples) of fruit flies among commonly cultivated cucurbit crops is limited particularly in Tanzania.

To address this knowledge gap, we collected fruit samples from three cucurbit species. The primary objective of this study was to identify the species of fruit flies infesting cucurbit crops. We rigorously test the hypothesis that infestation levels (the percent and degree of infestation) vary significantly among fruit fly species, agroecological zones and the host plants they attack. The findings from this study will provide valuable insights for developing an effective fruit fly management program in Tanzania.

## 2. Materials and methods

### 2.1. Study site

The study was carried out at two distinct altitudes of the Morogoro region referred to as agroecological zones, namely the “plateau zone” (low altitude) and the “mountainous zone” (high altitude) [17]. The Morogoro is situated in a transitional belt, falling between the coordinates of S 5°58’ – S 10°0’ latitude and E 35°25’ - E 38°30’ longitude [9]. The two zones differ in climatic characteristics and are at least 8 km apart. The plateau zone is located between 300–600 m above sea level, experiencing an annual rainfall ranging between 700–1200 mm and an average annual temperature of 29 ˚C [36–38]. This zone predominantly consists of conventional agriculture, cultivating a variety of horticultural crops, including, but not limited to, mangoes, citrus (oranges, lemons), eggplants, cucurbits (cucumbers, watermelons, and squash), and tomatoes. On the other hand, the mountainous zone is situated beyond 600 m above sea level, with an annual rainfall of 800–2500 mm and an average yearly temperature of around 24 ˚C [9,38]. It is characterized by agroecological farming practices and cultivation of a variety of crops, such as strawberry, cucurbits (watermelon and cucumber), maize, passion fruit, avocado, and banana. The mountainous area is situated close to the semi-natural forest of Uluguru mountain, where a wide range of wild fruits are also found throughout the year.

The choice of these two agroecological zones was also influenced by their well-known cucurbit production activities, making them ideal sites for fruit fly infestation studies [18,36,37]. Five locations, approximately 1 km apart, were identified within each agroecological zone (Table 1). In each location, three plots were established, each measuring 1120 m^2^ (70 m x 16 m). In plot 1, cucumbers (*Cucumis sativus* L.), specifically the “Ashley” variety, were planted with a 50 cm x 60 cm spacing. In plot 2, watermelons (*Citrullus lanatus* [Thunb.] Matsum. & Nakai.), the “Sugar baby” variety, were planted with a spacing of 1 m x 1.5 m. In plot 3, squash (*Cucurbita moschata* D.), the “Waltham” variety, was planted with a spacing of 1 m x 1.5 m.

**Table 1:** Locations of experimental plots in two agroecological zones of the Morogoro region, Tanzania.

Agroecological zones (AEZ)	Field name	Coordinates	Altitude
**Plateau**	SUA Horticulture Unit (HT)	S06˚50’41.4“ E37˚39’43.3“.	524 m
	SUA Crop Museum (CM)	S06˚51’00.53“ E37˚39’17.90“.	528 m
	SUGECO (SG)	S06˚50’22“ E37˚38’42.2“.	511 m
	SUA Mazimbu (MZ)	S06˚47’26.208“ E37˚38’7.926“.	486 m
	SUA Mafiga (MF)	S06˚50’22.764“ E37˚37’53.46“.	503 m
**Mountainous**	Morning Site (MS)	S06˚53’17.9“ E37˚40’14.93“.	1274 m
	Mkumbulu (MK)	S06˚52’24.2“ E37˚40’21.5“.	1105 m
	Ruvuma (RV)	S06˚52’34.6“ E37˚40’3.7“.	1000 m
	Kifuru (KF)	S06˚53’32.1“ E37˚40’9.5“.	1418 m
	Mgola (MG)	S06˚51’41.4“ E37˚40’4.3“.	1084 m

In all zones, the cucurbit species were cultivated over three consecutive seasons from March to October 2020. The seasons included the March–April season (referred to as Season I), the June–July season (referred to as Season II), and the September–October season (referred to as Season III). The March–April season was the early rainy season with an average rainfall of 222.3 mm, an average temperature of 30.5 °C, and a relative humidity of 92%. The June–July period represented the late rainy season, characterized by an average rainfall of 16.9 mm, an average temperature of 27.6 °C, and a relative humidity of 88.5%. Furthermore, the September–October season represented the dry season, with an average rainfall of 1.95 mm, an average temperature of 30.5 °C, and a relative humidity of 83% (see for detail in S1 Table).

### 2.2. Experimental design

The study used a randomized block design with five replications. It considered host species (Cucumber, Watermelon, and Squash) and fly species (five species) as factors. The seasons (March-April, June-July, and September-October) were only used as repetition periods of the study. The study adopted similar management practices in both the mountainous and plateau zones. These practices were standardized based on the harmonized practices commonly used by farmers in the two study sites in the Morogoro region to ensure consistency in the experimental setup.

### 2.3. Sampling methods and rearing of fruit flies

On a weekly sampling protocol, we harvested ten fruits per crop species for five consecutive weeks when the crops were at least 30% fruits set in a period spanning from March to October 2020. A set of ten fruits per crop species was collected destructively as a sample by randomly harvesting a mixture of immature and semi-matured fruits still attached to the crops. Samples were transported to the rearing facility at the Sokoine University of Agriculture (SUA) in Morogoro. Each sample was washed, weighed and placed in plastic incubation containers (24.8 cm × 16 cm ×13 cm) and observed at room temperature of 23–25°C following the method described by Copeland et al. [39]. Sterilised sand was placed at the bottom of the containers as pupation media. After 5 days, the sand was sifted daily and recovered pupae were placed on a moist filter paper in a petri dish. The petri dishes with pupae were then placed in population cages made of perspex (24.8 cm × 16 cm ×13 cm). Emerged adults were fed with an artificial diet containing enzymatic yeast hydrolysate (Natural Vitaminor^TM^, Natural Granen, Belgium) and sucrose (sugar) in a ratio of 1:3 [7]. Water was provided in petri dishes containing pumice granules for safe landing. After three days, adults were removed and killed using acetyl acetate following methods described by White and Elson-Harris [40]. The specimens were preserved in 98% absolute ethanol for morphological identification. The flies were identified under a microscope using keys and characters presented by White and Elson-Harris [41], White [42], Ekesi and Billah [42] and electronic keys by Virgilio et al. [43]. Some specimens were sent to the Royal Museum for Central Africa (RMCA) for further identification and confirmation.

### 2.4. Data collection

The number of positive and negative samples per crop species and the number of fruit fly individuals that emerged per crop species sample were counted and recorded based on the week the sample was collected.

### 2.5. Ethics statement

This study protocol was approved by the Ethics Review Board of the College of Agriculture, Sokoine University of Agriculture, Tanzania (Approval Number: MCS/D/2018/0023/11). Written informed consent was waived by the Ethics Review Board, as the study was conducted entirely within the land owned by Sokoine University of Agriculture.

### 2.6. Data analysis

#### 2.6.1 Percent infestation.

Percent infestation is the number of samples infested by fruit flies over the total number of samples collected per crop species. It provides insight into the severity of fruit fly damage on a particular crop and allows quantification of infestation levels [44]. The index was chosen to assess the probability of fruit fly infestation across different cucurbit species commonly grown in the Morogoro region. It further gives insight into potential monetary losses caused by fruit flies in export trade. In this study, a “positive sample” indicated the presence of at least one fruit fly individual.

The percent infestation was calculated using the following formula: -


$$\text{Percent Infestation }\left(\text{\%}\right)=\frac{\text{Number of samples infested by fruit flies}}{\text{Total number of samples collected }}\times 100$$ 1

To evaluate significant differences in percent infestation across fly species, crop species, and agroecological zones, we employed an Analysis of Variance (ANOVA) within the framework of generalized linear mixed-effects models (GLMMs) with binomial distribution to account for the proportional data (bounded between 0 and 1). The ANOVA for the model was performed using the Anova() function in the ‘car’ package. The model treated fruit fly species, crop species, and agroecological zones (study areas) as fixed effects, while seasons and field names were incorporated as random effects.

Due to initial model convergence issues, we first addressed potential problems related to singularity and gradient convergence. Model convergence was ultimately achieved by implementing the ‘bobyqa’ optimization method available in the glmerControl function of the lme4 package. Model validation involved examining residual plots against fitted values and predictors following the recommendations of Zuur et al. [45]. We estimated marginal means using the emmeans package to compare differences among fly species, crop species, and agroecological zones. To account for multiple comparisons, *P-*values were adjusted using the Tukey method.

#### 2.6.2 Degree of infestation(DOI).

The degree of Infestation is the number of adult fruit fly individuals emerge per kilogram (kg) of fruits. It provides a direct measure of the concentration of fruit fly larvae within a crop [44]. This index provides a measure of the host’s suitability. The index could be a function of, among others, the abundance of fruit flies and the availability of hosts (see Follett et al. [22]) and management practices. In this study, the DOI was employed to assess the relative suitability of different cucurbit species for a given fruit fly species. Higher DOI values are indicative of a cucurbit species’ higher vulnerability to fruit fly infestation, implying that the species offers an environment conducive to the pest’s reproduction and higher larvae performance. Conversely, lower DOI values suggest a reduced susceptibility of the cucurbit crops to fruit fly invasion, thereby signalling a potentially less favourable breeding ground for the flies. Thus, the DOI in the current study was used to predict the vulnerability of different cucurbit species to fruit fly infestation across sites.

The weekly degree of infestation was determined using the following formula: -


$$\text{Degree of Infestation }\left(\text{adult flies/kg}\right)\text{=}\frac{\text{Number of adult fly individuals emerged}}{\text{Weight of fruits (kg)}}.$$ 2

The weekly degree of infestation values were log-transformed using the formula ln(x+1) to normalize the data prior to analysis. Significance differences in the degree of infestation levels among host plants, fruit fly species, and agroecological zones were evaluated using an Analysis of Variance (ANOVA) within the framework of linear mixed-effects models (LMMs) assuming a Gaussian distribution. The ANOVA was performed using the Anova() function from the car package.

In the LMMs, fruit fly species, crop species, and agroecological zones (study areas) were treated as fixed effects, while seasons, sampling weeks (dates), and field names were included as random effects to account for hierarchical variability in the dataset. Model validation was conducted in accordance with the recommendations of Zuur et al. [45]. Standardized residuals were examined against fitted values and predictors to ensure that model assumptions such as homoscedasticity and normality of residuals were met. The normality of residuals was verified using Normal QQ plots. Marginal means were estimated using the emmeans package to facilitate pairwise comparisons of infestation levels among fruit fly species, crop species, and agroecological zones. P-values for these comparisons were adjusted using the Tukey method for multiple comparisons tests. All statistical analyses were carried out using R statistical software, version 4.4.1 [46].

## 3. Results

### 3.1 Percent infestation

We collected a total of 450 samples, representing 4,500 fruits, across the two agroecological zones during the three cropping seasons (1 sample was equivalent to 10 fruits). Each zone contributed an equal number of samples (i.e., 225 samples). About 92.9% (418) of the total samples were infested by fruit fly species. Of these, 50.96% (213 samples) were collected from the plateau zone and the remaining 49.04% (205) were from the mountainous zone (Table 2).

**Table 2:** Number and percentage of total infested samples among hosts in the mountainous and plateau zones.

Agroecological zone	Crop species	TNF	TNS	TNSI
**Mountainous**	*Citrullus lanatus*	750	75	62(82.7%)
	*Cucumis sativus*	750	75	73(97.3%)
	*Cucurbita moschata*	750	75	70(93.3%)
	Total	2250	225	205
**Plateau**	*Citrullus lanatus*	750	75	73(97.3%)
	*Cucumis sativus*	750	75	69(92.0%)
	*Cucurbita moschata*	750	75	71(94.7%)
	Total	2250	225	213
**GRAND TOTAL**		**4500**	**450**	**418 (92.9%)**

TNF: Total number of fruits collected, TNS: Total number of samples, TNSI: Total number of samples infested

**Fig 1:**
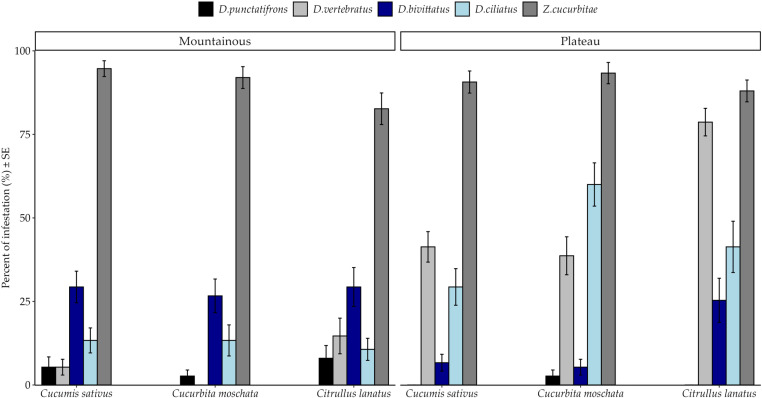
Percent Infestation of fruit fly species among cucurbit species across different agroecological zones of the Morogoro region from March to October 2020. SE stands for Standard Error.

**Fig 2:**
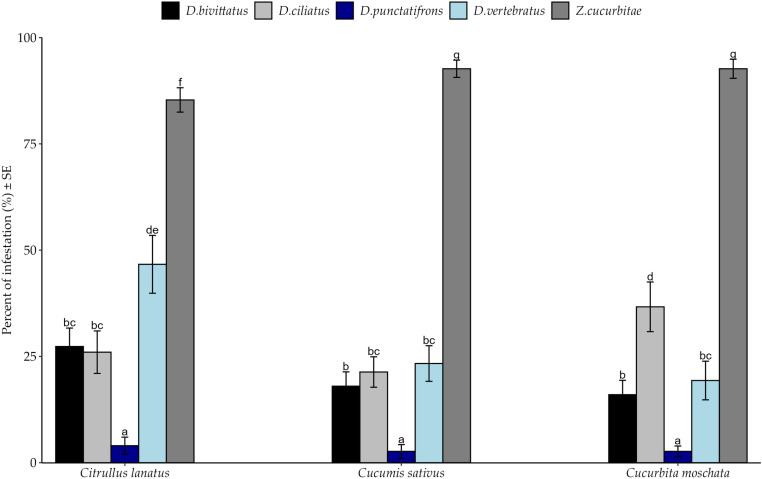
Percent Infestation of fruit fly species among cucurbit species in the Morogoro region from March to October 2020. Bars with different letters denote significant differences, p < 0.05. SE stands for Standard Error.

General percent infestation pattern shows dominance of *Z. cucurbitae* among hosts in both agroecological zones *(*Fig 1*).* However, the effects of thrree-way interactions were no significant. Percent infestation by fruit fly species varied significantly among crops (Table 3). *Zeugodacus cucurbitae* infested significantly more samples of the three hosts than other fly species at both agroecological zones (Fig 1). On the contrary, all cucurbit species were least attacked by *D. punctatifrons*(Fig 1). *Citrullus lanatus* and *C. moschata* were significantly more infested by *D. vertebratus* and *D. ciliatus* respectively than to other *Dacus* species (Fig 2). Susceptibility of *C. sativus* to *D. ciliatus*, *D. vertebratus* and *D. bivittatus* was statistically similar (*Post hoc* Test = Tukey HSD)*.*

**Table 3:** Analysis of Variance (ANOVA) based on GLMMs framework on effects of crop species (CS), fruit fly species (Fly) and Agroecological zone (AEZ) on percent infestation. Degree of freedom (df), Chi-square and p-values are displayed.

Source of Variations	Statistics		
***df***	***Chisq***	***P-value***
**Crop Species (CS)**	3	19.814	0.0002*
**Fruit fly Species (Fly)**	6	382.317	<0.0001*
**Agroecological zone (AEZ)**	2	23.419	<0.0001*
**CS × Fly**	8	44.022	<0.0001*
**Fly × AEZ**	4	102.329	<0.0001*
**CS × AEZ**	2	9.712	0.0078*
**CS × Fly × AEZ**	8	6.391	0.6035ns

**Fig 3:**
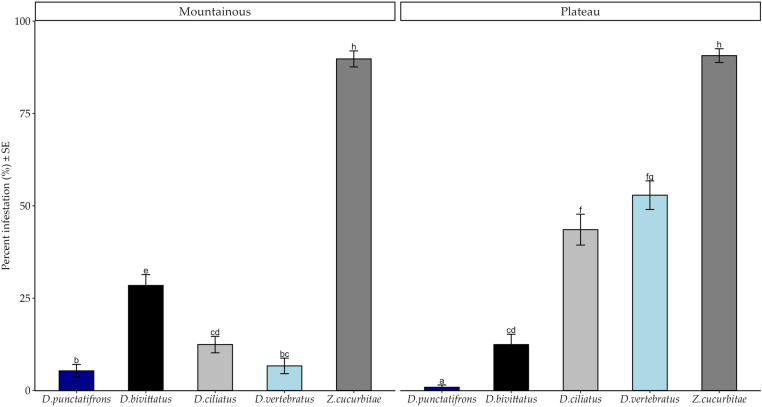
Percent Infestation of fruit fly species in the two agroecological zones of the Morogoro region from March to October 2020. Bars with different letters denote significant differences, p < 0.05. SE stands for Standard Error.

We recorded significantly higher infestation by *Z. cucurbitae* at both zones (Fig 3), followed by *D. vertebratus* and *D. ciliatus* at the Plateau zone, and *D. bivittatus* at the Mountainous zone. Percent infestations of *D. ciliatus* and *D. vertebratus* were significantly higher at the Plateau than at the Mountainous zone, in contrast to *D. bivittatus* and *D. punctatifrons* (Fig 3). The differences in percent infestation by *Z. cucurbitae* between the two agroecological zones were not significant (*Post hoc* Test = Tukey HSD)*.*

**Fig 4:**
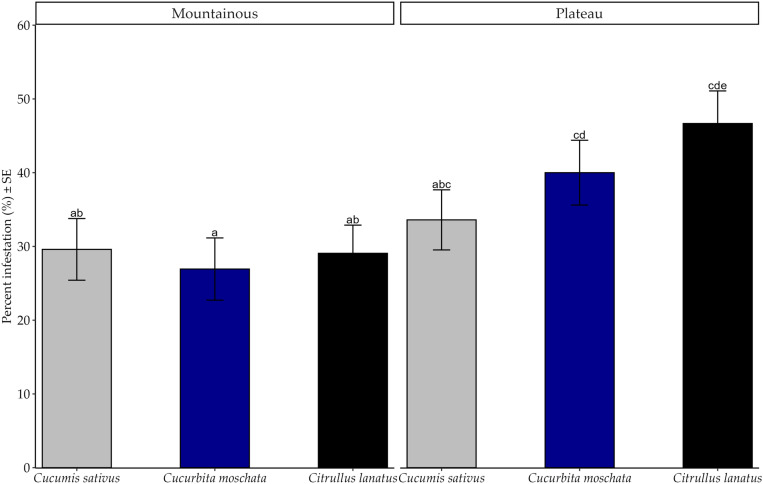
Percent Infestation of samples cucurbit species in the two agroecological zones of the Morogoro region from March to October 2020. Bars with different letters denote significant differences, p < 0.05. SE stands for Standard Error.

*Citrullus lanatus* fruits grown at the Plateau zone were significantly more susceptible to fruit fly attacks (Fig 4). However no significant variation among hosts was recorded at the Mountainous zone (Fig 4).

### 3.2 Degree of infestation

In total, about 22,169 fruit flies were recovered from three cucurbit species. Of these, 12,390 flies emerged from samples collected from the mountainous zone, while the remaining 9,779 flies (44.1%) emerged from samples collected from the plateau zone (Table 4).

**Table 4:** Overall number of flies that emerged among host species across agroecological zones.

Fly species	Plateau			Mountainous		
*C. lanatus*	*C.sativus*	*C. moschata*	*C. lanatus*	*C. sativus*	*C. moschata*
***Z. cucurbitae***	1932	2080	2907	2575	4830	4465
***D. vertebratus***	1657	149	162	56	11	0
***D. ciliatus***	190	143	451	26	32	49
***D. bivittatus***	69	19	18	81	126	106
**D. *punctatifrons***	0	0	2	25	6	2
**Total no.flies/crop**	**3848**	**2391**	**3540**	**2763**	**5005**	**4622**
**Total no. flies/zone**	**9779**			**12390**		
**Total no. of flies**	**22 169**					

**Fig 5:**
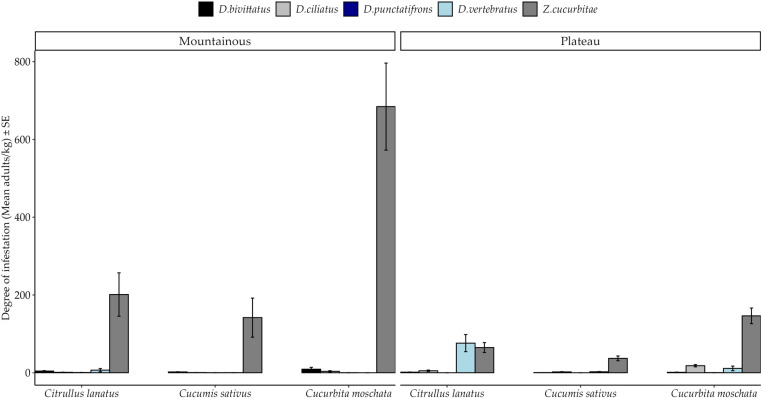
Degree of Infestation of fruit fly species among cucurbit species across the agroecological zones of the Morogoro region from March to October 2020. SE stands for Standard Error.

**Fig 6:**
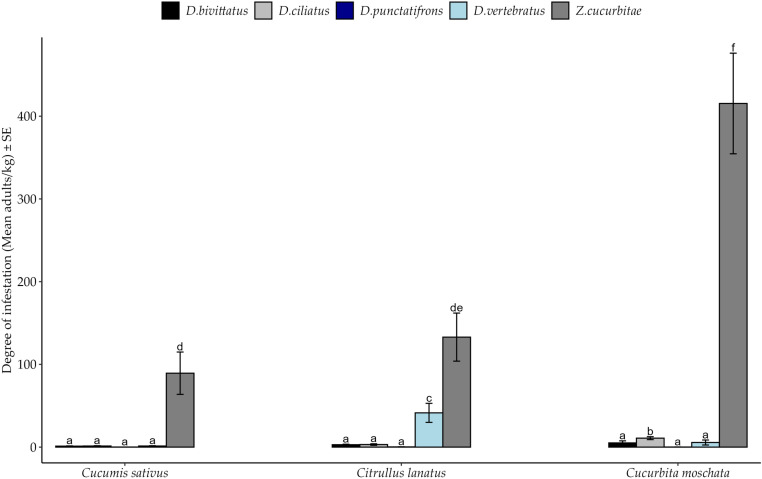
Degree of Infestation of fruit fly species among cucurbit species in the Morogoro region from March to October 2020. Bars with different letters denote significant differences, p < 0.05. SE stands for Standard Error.

Spatial pattern of degree of infestation by fruit fly species among hosts is presented in Fig 5. Generally *Z. cucurbitae* was the dominant species although, three-way interactions among factors had no significant effects. The degree of infestation by fruit fly species varied significantly between crop species (Table 5). In particular, the degree of infestation by *Z. cucurbitae* was significantly higher among all host species, followed by *D. vertebratus* in *C. lanatus* and *D. ciliatus* in *C. moschat*a (Fig 6). The degrees of infestation by remaining fly species were relatively low and without significant differences among hosts (*Post hoc* Test = Tukey HSD).

**Table 5:** Analysis of variance (ANOVA)based on LMMs framework on the effects of host plants (CS) fruit fly species (Fly) and agroecological zone (AEZ) on the degree of infestation. Degree of freedom (df), Chi-square and p-values are displayed.

Source of Variations	Statistics		
***df***	***Chisq***	***P-value***
**Crop Species (CS)**	3	72.359	<0.0001^*^
**Fruit fly Species (Fly)**	6	853.309	<0.0001^*^
**Agroecological zone (AEZ)**	2	0.266	0.8755ns
**CS × Fly**	8	97.958	<0.0001^*^
**Fly × AEZ**	4	156.304	<0.0001^*^
**CS × AEZ**	2	0.172	0.9178ns
**CS × Fly × AEZ**	8	6.756	0.563ns

* Indicates significance at alpha = 0.05 and **ns** indicates not significance

**Fig 7:**
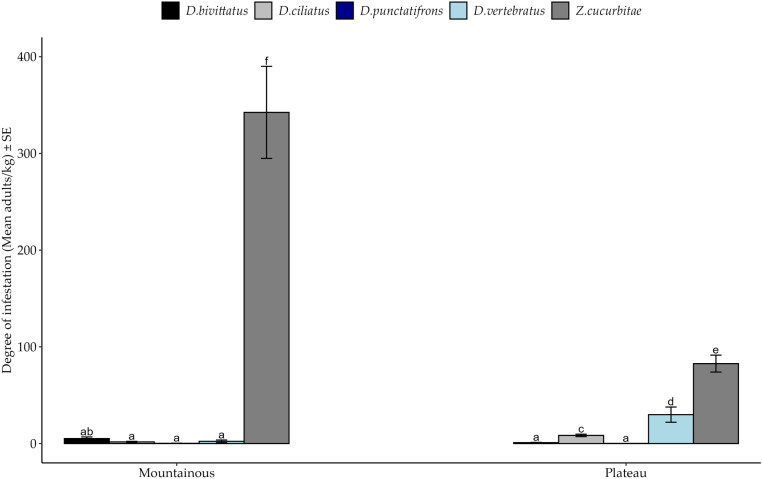
Degree of infestation of fruit fly species infesting cucurbit species at the two agroecological zones in the Morogoro region from March to October 2020. Bars with different letters denote significant differences, p < 0.05. SE stands for Standard Error.

Agroecological zone significantly affected the degree of infestation among fruit fly species (Table 5). Significantly higher degree of infestation of *Z. cucurbitae* than other species was observed at both zones, followed by *D. vertebratus* only at the Plateau zone (Fig 7). We further noted a significantly higher degree of infestation by *Z. cucurbitae* in the Mountainous than the Plateau zone, in contrast to *D. vertebratus.* The remaining fruit fly did not exhibit significant variations in the degree of infestation at the two agroecological zones (*Post hoc* Test = Tukey HSD)*.*

## 4. Discussion

Our study recorded five fruit fly species namely *Z. cucurbitae D. vertebratus, D. ciliatus, D. bivittatus*, and *D. punctatifrons* infesting cucurbits in the study region*.* The latter two species were recorded in low numbers, and have gained less research attention so far. Previous studies reported *Z. cucurbitae*, *D. vertebratus and D. ciliatus* as major infesters of cucurbits in various regions [12,18]. For example, Mwatawala et al.[18] reported a 72% infestation by *Z. cucurbitae* in the same study region in Tanzania. Similarly, Amin et al. [47] recorded a 71.5% infestation by *Z. cucurbitae* in Bangladesh. A study by Kambura et al. [12] found over 70% infestation of cucurbit hosts by *D. ciliatus* in Kenya while Am et al.[5] reported percent infestation of up to 73.83% by *D. ciliatus* in India. Additionally, Layodé et al. [48] in Benin reported an infestation rate of up to 145.5 flies/ kg in cucurbits. These findings highlight the significant threat posed by *Z. cucurbitae D. ciliatus* and *D. vertebratus* in cucurbit production.

We found significant dominance of *Z. cucurbitae* among hosts at the two agroecological zones. *Zeugodacus cucurbitae* had a higher percent infestation and degree of infestation than the other four infesters in all fruits at both agroecological zones. Comparative studies on fruit infestation by the exotic *Z. cucurbitae* and native African species are limited and results vary with region. Previous studies in East Africa reported *Z. cucurbitae* as the main pest of cucurbits with higher incidences and infestation rates than the native species, except in a few cases [12,18]*.* Reports from West Africa showed dominance of *D. vertebratus* over *Z. cucurbitae* and other *Dacus* species [35,48]. A study by Mokam [3] from the same region reported dominance of *D. bivittatus* and *D. ciliatus* over *D. vertebratus* in studied hosts. Unfortunately, the available information on cucurbit infesters is based on limited number of observations and requires further extensive sampling [19] before a firm conclusion can be made.

Observed variations in percent and degree of infestation could be linked to the history of the introduction of the exotic species and host specilization. *Zeugodacus cucurbitae* is an exotic, invasive species of Asian origin that was introduced in Africa [40] with the first record from Tanzania, East Africa in 1936 [30,31]. Its presence in West Africa is only confirmed since the beginning of the 21^st^ Century [30]. *Zeugodacus cucurbitae* has co-existed in East Africa for a much longer time than in West Africa. Fruit fly species often develop host specialization through long-term coevolutionary interactions with their preferred host plants [49,50]. These interactions can lead to the evolution of specialized physiological and behavioral traits, enabling fruit fly species to utilize a wide range of host plants for oviposition and larval development [14,51,52]. Thus variations in the degree of infestations among coexisting fruit fly species may also be attributed to evolutionary adaptations that have allowed these species to exploit specific host plants that offer optimal nutritional profiles for their development and fecundity [53].

Competition is another factor that may contribute to dominance of *Z. cucurbitae* over native Dacus species. *Bactrocera* and *Zeugodacus* species are best adapted to exploit and to compete with other species in the same ecological niche [54]. *Zeugodacus cucurbitae* is an important competitor with indigenous fruit fly species while being predominant in most Cucurbitaceae [30]. The species has higher reproductive potential and lower developmental thresholds [7,26,55]. These biological advantages endow *Z. cucurbitae* with a competitive advantage over other native species, contributing to its dominance within the region [7,18,56]. According to Vayssières et al. [7] larval development parameters of *Z. cucurbitae* can give this species an advantage over *D. ciliatus* in case of larval exploitative competition. High infestation rate of *Z. cucurbitae* could thus be attributed to its demographic strategy, exploitative competition and larval interference [34]. Studies on the interspecific interactions between these tephritid fruit flies on vegetables in Africa are scarce [30]. Further studies on the interspecific interactions between these cucurbit feeders in Africa is crucial to elucidate the extent of this competition [12].

Variations in percent infestation and degree of infestation of fruit flies between agroecological were significant, but slight, with an exceptional case of degree of infestation of *Z. cucurbitae* that was the most dominant. The higher infestation of *Z. cucurbitae* in the Mountainous zone could not be further explored by this study, since the species is known to be a low altitude resident. Likewise the variations in percent infestation among hosts between the two agroecological zones were only significant at the Plateau zone.

In our case, infestation among hosts by pests did not vary significantly, with a few exceptions. Susceptibility of cucurbit hosts can be affected by morphological and biochemical defense mechanisms of fruits [5,57,58]. For example, high contents and activities of antioxidants and defense enzymes as well as hardness, thickness of the rind and pubescence on the skin may reduce infestation by fruit flies [5]. However, our study did not specifically evaluate the morphological and biochemical properties of the studied hosts, and we recommend further studies in this area. These results conclude that the interaction between the host and fruit flies species is key in modulating percent infestation and degree of infestation. This implies further that the two agroecological zones of the Morogoro region are within the suitable climatic ranges of the studied cucurbit infesters. However, the design and duration of the study could not allow rigorous analysis to determine the contribution of abiotic factors and some biotic factors to the percent and degree of infestation. Future studies should also encompass a broader range of potential hosts that have been identified in prior studies to provide a more comprehensive overview of host preferences and infestation dynamics among fruit flies in this region.

## 5 Conclusion

This study conclusively establishes the prevalence and destructive impact of *Zeugodacus cucurbitae*, a primary pest, along with other significant fruit fly species such as *Dacus vertebratus*, *Dacus ciliatus*, *Dacus bivittatus*, and *Dacus punctatifrons*, on cucurbit crops in the Morogoro region of Tanzania. The severity of infestations underscores the urgent need for the implementation of effective fruit fly management practices to safeguard cucurbit production in this area. This research suggests that agroecological management strategies adopted by farmers should prioritize the control of *Z. cucurbitae* as a key pest, especially considering its association with key host species such as *Cucumis sativus* and *Cucurbita moschata*. By focusing on the ecological interactions between these pests and their preferred hosts, farmers can adopt more targeted and sustainable pest control measures. This study contributes valuable insights into the pest dynamics within cucurbit agricultural systems, offering a foundation for developing integrated pest management (IPM) strategies tailored to the specific conditions of Tanzania and tropical Africa. The knowledge gained from this research is instrumental for agricultural stakeholders, including farmers and extension workers, in devising and implementing effective measures to combat fruit fly infestations, thereby enhancing the productivity and profitability of cucurbit farming in the region.

## Supporting information

S1 Table Weather data. The weather variable data on temperature, rainfall and relative humidity was collected during the study in the Morogoro region from March to October 2020.
(XLSX)

S2 Table The minimal data set for the Degree of Infestation (DOI). The data was collected during the rearing of the fruit flies infested cucurbit crops in the Morogoro region from March to October 2020.
(XLSX)

S3 Table The minimal data set for Percent Infestation (PI). The data was collected during the rearing of the fruit flies infested cucurbit crops in the Morogoro region from March to October 2020.
(XLSX)

S4 Table The metadata for the study. The data was collected during the rearing of the fruit flies infested cucurbit crops in the Morogoro region from March to October 2020.
(XLSX)
