# Correction: Fruit fly infestation of cucurbitaceous vegetables in Morogoro—Eastern Central Tanzania

**DOI:** 10.1371/journal.pone.0325451

**Published:** 2025-05-28

**Authors:** The PLOS One Staff

## Notice of Republication

This article was republished on May 14, 2025, to correct an error in the article PDF. The citation in the article PDF was incorrect due to a typesetting error. The publisher apologizes for this error.

## References

[1] Tarimo P, Kabota S, Majubwa R, Kudra A, Virgilio M, Jordaens K, et al. Fruit fly infestation of cucurbitaceous vegetables in Morogoro-Eastern Central Tanzania. PLoS One. 2025;20(4):e0322277. doi: 10.1371/journal.pone.0322277 40258002 PMC12011243

